# Small Bowel Obstruction by a Phytobezoar in a Patient With Previous Antrectomy and Billroth II Reconstruction

**DOI:** 10.7759/cureus.45849

**Published:** 2023-09-24

**Authors:** Alberto Abreu da Silva, Jéssica Ricardo, Andreia Ferreira, Diogo Sousa, José Augusto Martins

**Affiliations:** 1 General Surgery, Hospital do Litoral Alentejano, Santiago do Cacém, PRT

**Keywords:** enterotomy, small bowel obstruction, phytobezoar, intestinal obstruction, bezoar

## Abstract

A phytobezoar is a conglomerate of improperly digested fruit and vegetable debris, and its development is associated, amongst other factors, with previous gastric surgery. Most phytobezoars remain asymptomatic and are incidentally found during imaging or interventional procedures. However, in some patients, they can cause small bowel obstruction, which can subsequently lead to severe complications. Although the clinical findings are similar to other causes of intestinal obstruction, there are some particular diagnostic and treatment features more specific to phytobezoars. We present a case of an 85-year-old man with a history of previous antrectomy and Billroth II reconstruction who came to the emergency department with bilateral aspiration pneumonia and intestinal obstruction due to a bezoar. The CT scan showed bilateral inferior lobe pulmonary consolidation, as well as a marked dilation of the small bowel with gas-fluid levels and a transition to normal caliber in the terminal ileum, where an oval mottled-appearing mass suggesting a bezoar was present. An urgent laparotomy confirmed the diagnosis, and an enterotomy with removal of the bezoar was performed. Phytobezoars must be considered as a cause of intestinal obstruction, particularly when patients have a history of previous gastric surgery. Its radiological findings, particularly in CT scans, are specific and should be appreciated to establish the diagnosis promptly. The treatment of small bowel obstruction due to a phytobezoar requires surgery most of the time, and the surgeon must bear in mind the need to look for the existence of other bezoars in the gastrointestinal tract to prevent reoccurrence.

## Introduction

Bezoars are unproperly digested conglomerates that form in the gastrointestinal tract, more frequently in the stomach. While many materials can cause bezoars, the most frequent are phytobezoars, which are made of undigested parts of fruits and vegetables [1,2]. Most bezoars remain asymptomatic and are incidentally found in imaging studies or during endoscopic or surgical procedures [3]. However, some cases can become symptomatic. Bezoars have been associated with abdominal pain, intestinal obstruction, or gastrointestinal haemorrhage, usually a result of a bleeding gastric ulcer caused by the compression of the bezoar on the gastric mucosa [2]. When suspecting a bezoar, it is important to detail the patient’s past medical history since predisposing factors such as previous gastric surgery, inflammatory bowel disease, or gastrointestinal tumours might increase suspicion of the diagnosis [4]. We present a rare case of small bowel obstruction by a phytobezoar causing lethal systemic complications.

## Case presentation

**Figure 1:**
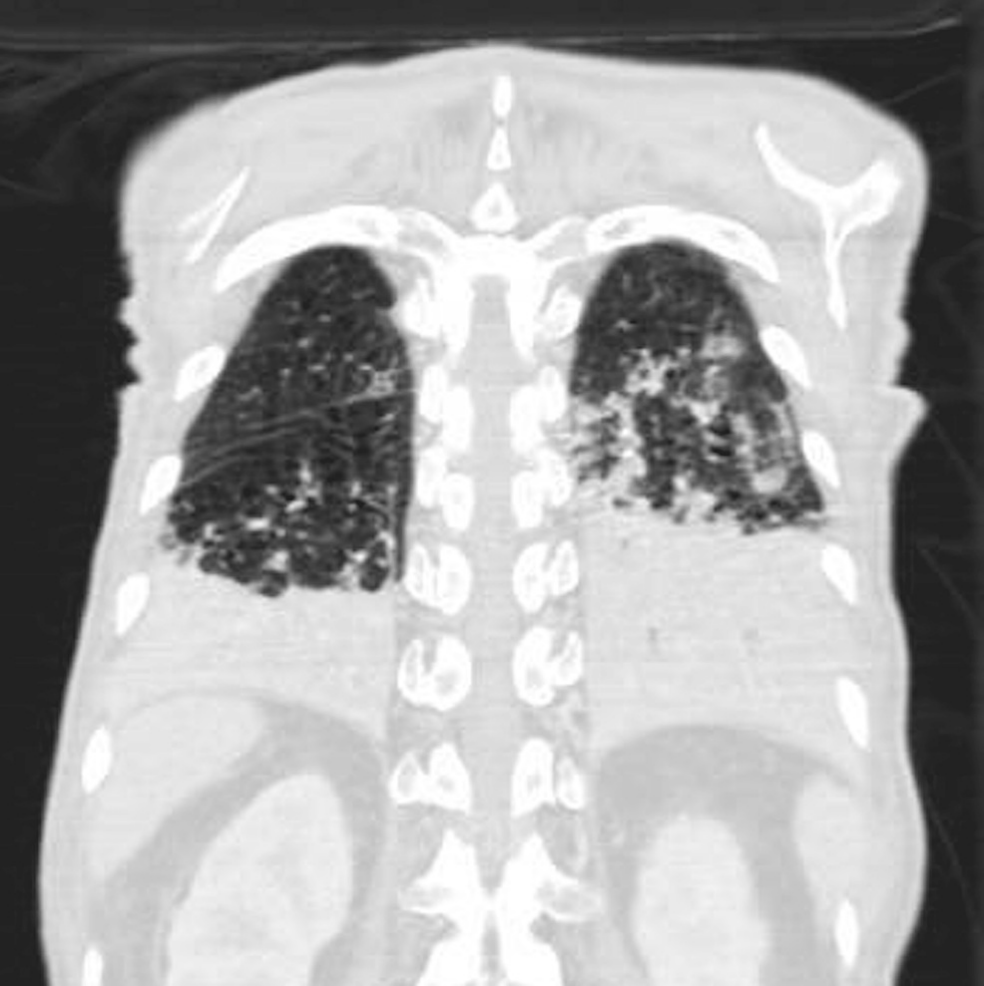
Coronal contrast-enhanced chest CT scan showing bilateral pulmonary inferior lobe consolidation.

**Figure 2:**
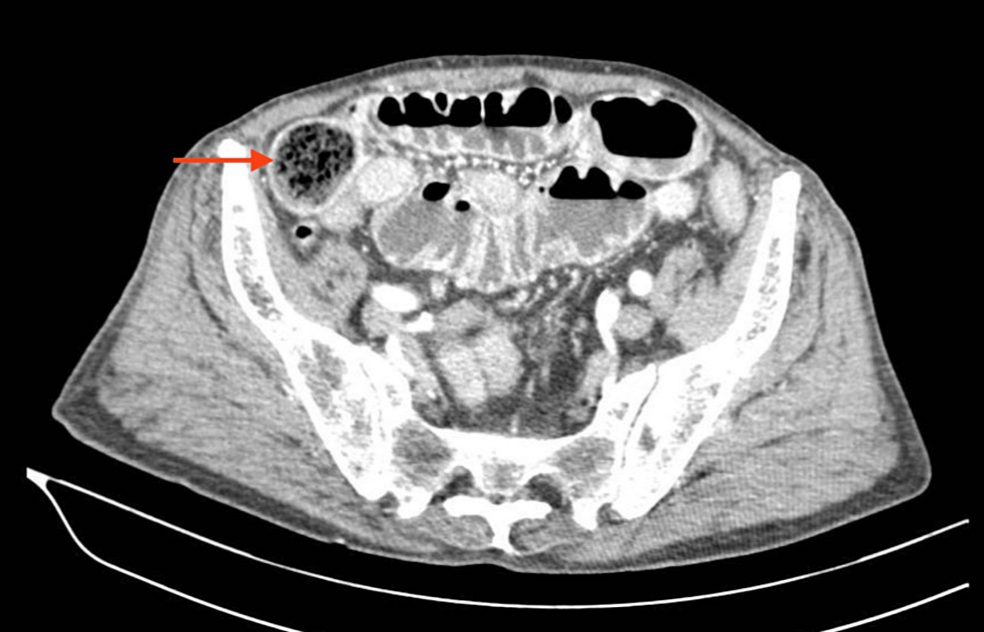
Axial contrast-enhanced abdominal CT scan revealing an oval mottled-appearing mass suggesting a bezoar (arrow), with proximal small bowel distension.

An 85-year-old man, generally healthy and independent, presented to the emergency department with severe abdominal pain for the last 24 hours, vomiting, diffuse abdominal distension, and shortness of breath. He previously underwent antrectomy with Billroth II reconstruction due to a perforated gastric ulcer 40 years ago. He also had diabetes, treated with oral antidiabetic agents, and chronic obstructive pulmonary disease due to heavy smoking. On admission, he was tachycardic, with normal blood pressure, febrile up to 38.4 C degrees, had a peripheral oxygen saturation of 84% on a high flow mask, and consciousness impairment to Glasgow Coma Scale of 9. He was using accessory respiratory muscles and on auscultation had diminished breath sounds with bilateral rhonchi and crackles. The abdomen was distended and diffusely tender with absent bowel sounds. A nasogastric tube was placed with an immediate release of 2 litres of faecal content. Blood tests revealed raised inflammatory markers and the arterial blood gas showed a metabolic acidosis with type 1 respiratory failure and a lactate concentration of 4.7 mmol/L. The patient was intubated, and after prompt resuscitation was taken for a CT scan. Images showed a bilateral inferior lobe consolidation secondary to gastric content aspiration (Figure 1), as well as a marked dilatation of the small bowel with gas-fluid levels and a transition to normal calibre in the terminal ileum, where an oval mottled-appearing mass of 52x32x30 mm suggesting a bezoar was visible (Figure 2).

**Figure 3:**
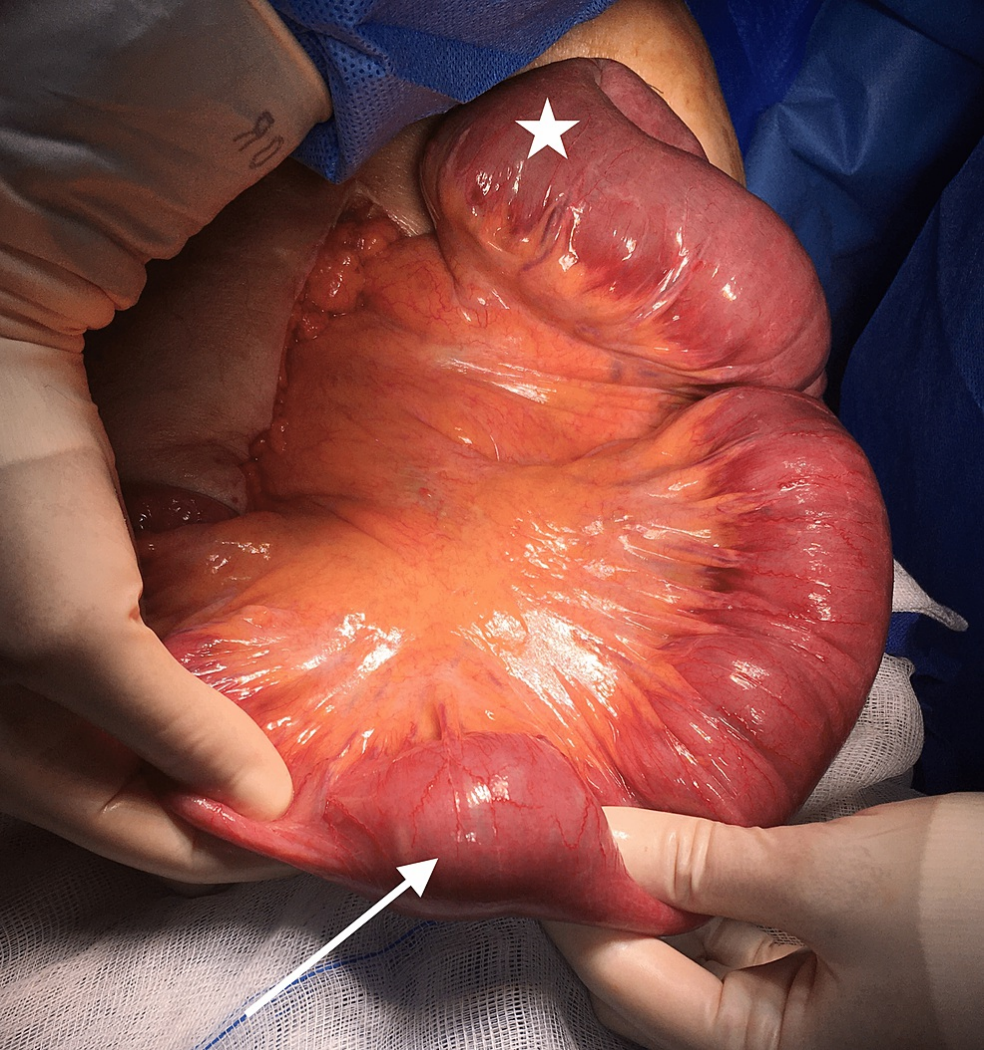
Intraoperative findings reveal an intraluminal mobile mass in the terminal ileum (arrow), with dilated proximal bowel loops (star).

Intestinal obstruction leading to bilateral aspiration pneumonia with septic shock was confirmed. We performed an urgent laparotomy, which revealed a small bowel obstruction caused by an intraluminal and mobile body (Figure 3).

**Figure 4:**
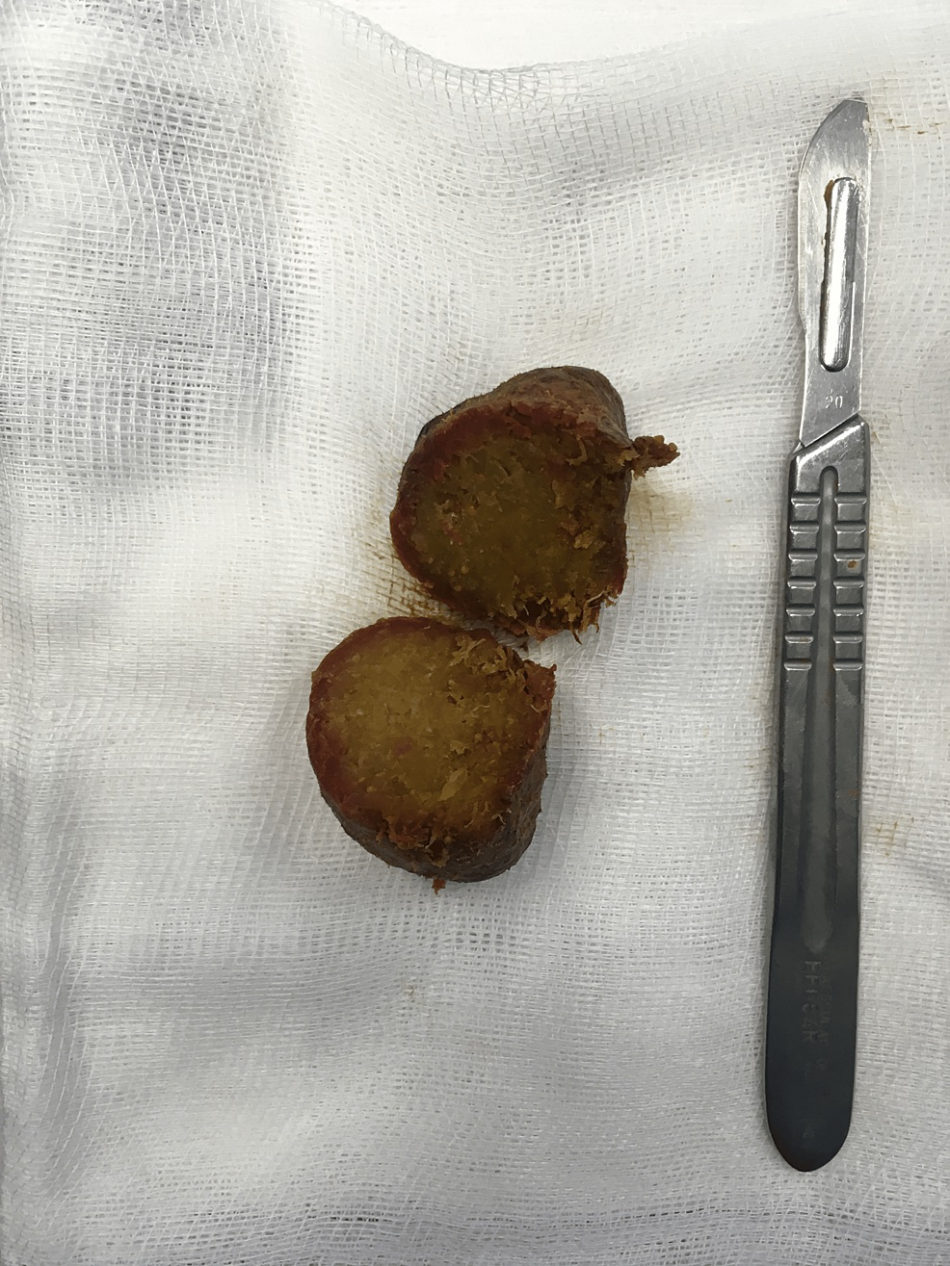
Phytobezoar specimen after removal and section.

We proceeded with an enterotomy, followed by extraction of a phytobezoar (Figure 4).

Before closing the enterotomy, we inspected the gastric remnant and the small bowel proximal to the obstruction site to exclude the existence of other bezoars. The patient was subsequently admitted to the intensive care unit. Unfortunately, bilateral pneumonia did not respond to treatment and on the 20th day after surgery, the patient passed away.

## Discussion

Bezoars, usually found in the stomach, are undigested conglomerates formed in the gastrointestinal tract by materials that are swallowed. Primary intestinal bezoars are much rarer and are associated with underlying small bowel diseases such as strictures, diverticula or tumours [1].

The most frequent type of bezoars are phytobezoars (made of compounds of fruits and vegetables), followed by trichobezoars (made of hair), pharmacobezoars (made of medications), and lactobezoars (made of milk protein) [2]. The occurrence of bezoars is rare, with a reported prevalence in endoscopic studies of 0.068% [3]. 

Several predisposing factors contribute to bezoar formation. It is reported that 42.8% of patients with bezoars had previous gastric surgery [4], such as partial gastrectomy, vagotomy and/or pyloroplasty, which ﻿alter the anatomy and physiology of the gastric remnant [5], and it is estimated that the incidence of bezoar formation following gastric surgery ranges from 5% to 12% [4]. It is postulated that delayed gastric emptying, with loss of normal pyloric function and gastric motility with low gastric acidity, increases the probability of bezoar development [6]. However, more recent studies suggest that this factor is not essential for bezoar formation, since it depends on multiple predisposing factors, such as the composition of the ingested materials [4]. Other reported predisposing factors are ﻿psychiatric or mental disease, peptic ulcer disease, chronic gastritis, Crohn’s disease, gastrointestinal tract tumours, dehydration and hypothyroidism. Also, bezoars have been associated with patients suffering from diabetic neuropathy ﻿and myotonic dystrophy [2], as well as with mastication problems and antacid drug use [4]. This patient had a previous antrectomy with a Billroth II reconstruction due to a perforated peptic ulcer 40 years before. This was confirmed intraoperatively and certainly contributed to changes in the gastric motility of the patient, which promoted the formation of the phytobezoar.

Most bezoars remain clinically silent. Although bezoars can give origin to unspecific abdominal pain or postprandial fullness, the most important presentations are gastrointestinal obstruction or bleeding [2]. Obstruction is more frequent in the small bowel, which accounts for 0.4-4% of all mechanical small bowel obstruction cases [7]. However, it can obstruct the gastric outlet and less frequently the colon. Bleeding is secondary to an ulcer, caused by pressure-induced necrosis of the gastric mucosa by the bezoar [2]. Our patient presented to the emergency room with severe respiratory failure following aspiration of high amounts of vomit due to an intestinal obstruction. Other complications of bezoars include intestinal perforation [8], intestinal intussusception [9], and obstructive jaundice in patients with biliodigestive anastomosis [10].

Intestinal obstruction by a bezoar has specific imaging findings. In plain abdominal films, besides the usual dilated small bowel loops with gas-fluid levels, if barium contrast is administered, it is possible to see an intraluminal filling defect, when barium fills the phytobezoar, which gives it a mottled appearance [11]. The CT findings are more specific since they can show an intraluminal ovoid or round mottled-appearing mass with soft tissue density containing air in its interstices and, if administered, outlined by fluid or oral contrast material [12]. Nonetheless, these features must be distinguished from the similar ﻿small-bowel faeces sign, which is a common finding in cases of progressive development of mechanic small bowel obstruction. In order to distinguish them, it has been proposed that if the mass is ovoid, its length is less than 10 cm and a similar gastric mass coexists, the likelihood of a bezoar being the obstructing cause would increase. However, a study suggested that a floating fat-density debris sign, which are images of fat-density debris floating in the lumen proximal to the obstruction, was more specific to distinguish a bezoar from the small-bowel faeces sign [13]. It has been proposed that bezoars mostly occlude the terminal ileum since this region is narrower, the motility is lower, and there is higher water absorption, which hardens the bezoar [14]. However, some articles report conflicting results, with more bezoars being found in the jejunum [11]. In the current case, it was possible to identify on the CT scan an oval intraluminal mottled-appearing mass with 52x32x30 mm in the terminal ileum that, together with the patient’s surgical history, increased suspicion of an obstructing bezoar.

The treatment of small bowel obstruction starts off with adequate fluid replacement and intestinal decompression with a nasogastric tube. If the position of the bezoar allows it to be reached by an endoscopic method, it can be treated with mechanical disintegration and/or chemical dissolution. However, definitive treatment is usually surgical. Fragmentation and squeezing out the bezoar can be primarily attempted. In case it is not possible, an enterotomy to allow removal of the bezoar should take place, which can be open surgery or laparoscopic [15]. Segmental enterectomy is indicated when there are signs of intestinal ischemia or perforation. During surgery, it is important that the surgeon excludes the existence of other bezoars in the gastrointestinal tract since it is common to find multiple bezoars, which can lead to a subsequent obstruction [7].

## Conclusions

Intestinal obstruction by a phytobezoar is a rare condition that can lead to devastating outcomes. This article showcases the importance of a detailed anamnesis when approaching a patient with intestinal obstruction. A patient with previous gastric surgery presenting with symptoms of intestinal obstruction should raise suspicion of a bezoar. Specific radiological findings, particularly the presence of a mottled-appearing mass at the obstruction point, can lead to early diagnosis and prompt management decisions. Finally, exploration during surgery to exclude the existence of other bezoars is essential in order to prevent further obstructions due to another bezoar left in situ.
